# Associating Parental Efficacy with the Utility of Smart Devices: A Cross-Sectional Study of Their Role in Alleviating Maternal Parenting Concerns among Infants Aged 6–11 Months

**DOI:** 10.3390/children10091437

**Published:** 2023-08-23

**Authors:** Ryuta Onishi, Hanami Tone, Minori Kubota, Nana Chino, Funa Maruyama

**Affiliations:** 1 Faculty of Nursing, Toyama Prefectural University, 2-2-78, Nishinagae, Toyama-shi 930-0975, Toyama-ken, Japan; 2 Faculty of Health Sciences, Hokkaido University, Kita 12, Nishi 5, Kita-ku, Sapporo-shi 060-0812, Hokkaido, Japan; 3 Department of Health Sciences, School of Medicine, Hokkaido University, Kita 12, Nishi 5, Kita-ku, Sapporo-shi 060-0812, Hokkaido, Japan

**Keywords:** parenting concerns, anxiety, smart devices, parental self-efficacy, child-rearing

## Abstract

In digital societies, the use of smart devices to solve childcare problems has become commonplace. Mothers are influenced both positively and negatively by smart devices used to resolve childcare concerns. Focusing on parental self-efficacy, this study identified the factors associated with relief and anxiety caused by the use of smart devices to eliminate parenting concerns among mothers with infants. A random sampling cross-sectional survey was administered to 257 Japanese mothers with infants aged 6–11 months. Structural equation modeling was used to explain the relief and anxiety caused by their use of smart devices in terms of maternal demographics, parental self-efficacy, smart-device dependence, and confidence in their ability to discriminate information. Mothers with high parental self-efficacy experienced increased relief and reduced anxiety by using smart devices to address concerns about child-rearing practices. Mothers who were highly dependent on smart devices felt more secure with their use of smart devices. Homemakers and highly educated mothers who used smart devices because of concerns regarding child health and development experienced more anxiety. Parenting professionals need to recognize the effectiveness of smart devices as a tool to relieve anxiety in parenting and provide additional support for parents to improve their parenting self-efficacy.

## 1. Introduction

### 1.1. Background

Parental anxiety, specifically within the context of infant and toddler rearing, poses a critical concern that not only endangers the psychological health of the parents but also potentially impedes the healthy development of the child. Manifestations of anxiety and depression can amplify parental stress and potentially escalate into significant risk factors for child maltreatment [1,2]. Moreover, a compelling correlation has been identified between parental anxiety and the incidence of anxiety disorders in their offspring [3,4]. Specifically, mothers of infants appear to be particularly susceptible to feelings of loneliness [5], with these feelings contributing to higher levels of anxiety around child-rearing due to psychological health implications [6]. Therefore, addressing the issue of parenting anxiety, especially in mothers at the heart of this predicament, is of importance.

Parents previously resolved child-rearing concerns by obtaining advice directly from the people around them or through child-rearing books. However, in today’s digital society, parents tend to resolve their child-rearing concerns by using devices such as smartphones or tablets (hereinafter “smart devices”). 

In the contemporary digital landscape, diverse parenting scenarios have seen a significant infiltration of digital resources [7,8]. A notable proportion of expectant mothers and those already embracing the role resort to the internet as a primary source of parenting information and guidance [9,10,11]. The burgeoning digital marketplace hosts an abundant supply of applications specifically designed to assist expectant mothers and those engaged in childcare, underlining the digital transformation in parenting approaches [12,13].

The integration of smart devices in parenting presents a spectrum of both positive and negative outcomes. On the positive side, the internet provides instantaneous and boundless access to contemporary parenting information [12,14], serving as a rich repository of knowledge. Furthermore, social networking sites (SNSs) offer a sense of community, reducing feelings of isolation by fostering connections among parents [15]. Thus, mothers utilizing SNSs may find themselves better equipped to navigate the complexities of childcare, knowing they are not alone in their struggles. Conversely, the abundance of information available online requires a certain degree of information literacy [14]. The inability to discern and select appropriate information can inadvertently foster anxiety, turning the benefits of digital accessibility into a source of distress. Moreover, the social comparisons enabled by SNSs can, in some cases, precipitate feelings of depression among mothers [16,17], as they gauge their parenting styles and successes against others. In essence, while smart devices can serve as a source of comfort in addressing childcare concerns, they may also contribute to the propagation of anxiety. Therefore, to fully harness the potential benefits of smart devices in alleviating childcare-related concerns, it is crucial to identify factors that can heighten a sense of security and reduce anxiety among mothers. 

Previous studies of smart-device use in parenting have primarily focused on their relationships with children’s health, growth, development, and life. Examples include studies examining the link between the screen time of parents and children [18] and the impact of mobile devices on parent–child interactions [19]. Studies of parental smart-device use in parenting are limited and include those on building social capital through information and communication technology use [20], social comparison through SNS use [16,17,21], and online parental interaction [7,22]. However, few studies have examined parents’ psychology when using smart devices to resolve parenting concerns. Therefore, this study identified factors associated with relief and anxiety resulting from smart devices’ use as a means of eliminating parenting concerns among mothers with infants.

### 1.2. Theoretical Framework

To develop a theoretical perspective on the behavioral patterns associated with using smart devices as a problem-solving tool in childcare, we drew upon various theories of information-seeking behavior. In presenting a model of information-seeking behavior, Wilson [23] underscored the intricate underpinnings of information search, which involve demographics, psychological predispositions, social roles, environmental factors, and characteristics of the information source. We anticipate that the outcomes of smart device usage are related to the mother’s demographics and contextual factors, such as how she employs these devices to tackle specific parenting challenges. Additionally, Wilson [23] highlighted the significance of self-efficacy in information-seeking behavior. Self-efficacy was defined as “concerned with people’s beliefs that they can exert control over their motivation and behavior and over their social environment” [24]. In the context of parenting, parental self-efficacy refers to a parent’s conviction that they can positively influence their child’s health and success [25]. Positive outcomes of parent–child relationships, child development, and parental mental health have been associated with parental self-efficacy, emphasizing its high clinical relevance [26]. A number of strategies have also been proposed to enhance parental self-efficacy [27,28]. Self-efficacy, viewed as a crucial factor in effective parenting and a potential intervention point, could significantly impact the outcomes of smart device usage. Thus, we focused on parental self-efficacy in the factors related to the relief and anxiety mothers experience due to the use of smart devices.

**Figure 1:**
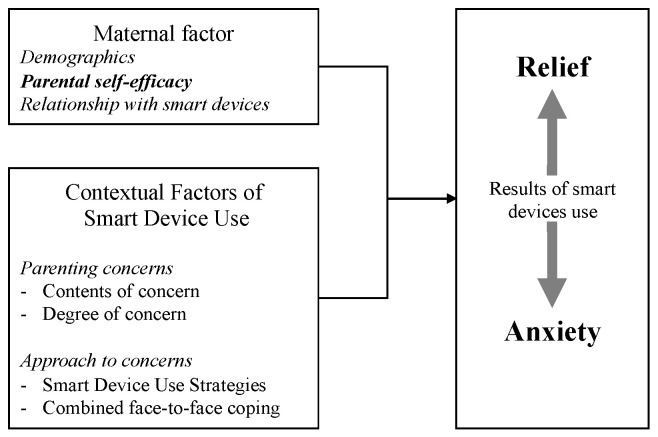
Theoretical framework.

Moreover, the technology acceptance model (TAM) posits that technology use is governed by perceived usefulness and ease of use [29]. We considered the possibility that a mother’s affinity for smart devices and the perceived utility of information obtained from these devices could affect device usage. Bearing these points in mind, we constructed a theoretical framework (Figure 1) suggesting that the results of smart device usage are influenced by maternal factors (demographics, parenting self-efficacy, relationship with devices) and contextual elements (factors of parenting concerns and approach to these concerns). Based on this framework, we explored the factors that promote the effectiveness of smart devices in resolving parenting concerns, focusing on parenting self-efficacy, which is a significant intervention point.

Therefore, in this scholarly inquiry, we propound three research queries: (1) To what degree does the utilization of smart devices to mitigate parental concerns furnish mothers with an ensuing sentiment of relief and anxiety? (2) In what way does a mother’s parenting self-efficacy relate to the effects of her use of smart devices to address parenting concerns? (3) How do ancillary maternal and contextual factors relate to the results of her engagement with smart devices to allay parenting concerns?

## 2. Methods

### 2.1. Study Design

A cross-sectional observational research design was adopted. The Checklist for Reporting of Survey Studies (CROSS), developed by Sharma et al. [30], was used to ensure quality and transparency in reporting.

### 2.2. Participants and Procedure

**Figure 2:**
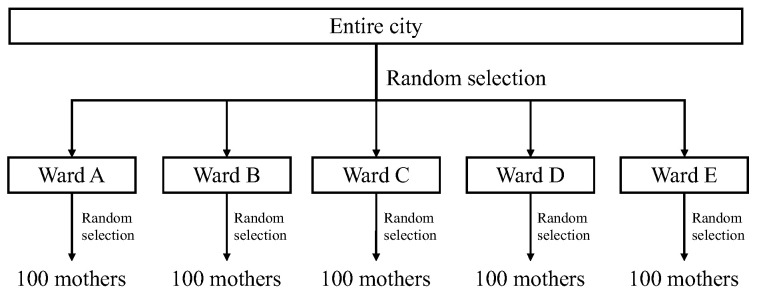
Stratified random sampling process.

Participants were 500 mothers living in Japan, each with a child aged between 6 and 11 months. Stratified random sampling was used (Figure 2). Five wards were chosen randomly from an urban region in Japan, and mothers with children aged between 6 and 11 months as of 1 June 2020, were randomly selected using the basic resident register of the wards. Data were collected using a mailed, self-administered questionnaire. This paper reports data from part of the “Using Smartphones to Address Childcare Concerns of Mothers with Infants” survey administered from June to July 2020.

### 2.3. Measures

The survey items in this study focused on participants’ demographics, parental self-efficacy, relationship with smart devices, and use of smart devices to eliminate parenting concerns. The content validity of the questionnaire was confirmed by a public health nurse with child-rearing support experience and two researchers with extensive public health nursing practice and research experience. The absence of common errors in questionnaires was confirmed by two researchers with extensive experience in questionnaire surveys. Additionally, the face validity of all survey items was confirmed through a preliminary survey of 12 mothers with infants and older children.

#### 2.3.1. Demographics

Information regarding participants’ age, number of children, occupational status, educational level, and subjective economic status was collected. Specifically, participants were asked to select one option that best described their current occupational status, with the choices being “Full-time workers”, “Part-time workers”, “Workers on maternity or childcare leave”, or “Homemaker”. Similarly, regarding educational level, participants were asked to choose from “Junior high school graduate”, “High school graduate”, “Junior college/Vocational school graduate”, or “University/Graduate school graduate”. Finally, to gauge their subjective economic status, participants were presented with four options: “Very concerned”, “Somewhat concerned”, “Slightly concerned”, and “Not concerned at all”.

#### 2.3.2. Parental Self-Efficacy

The Parenting Self-efficacy Scale [31] was used. This scale was developed in a Japanese context and has been tested for reliability and validity in a survey of mothers with infants [31]. It comprises 13 items, each of which is rated on a five-point Likert-type scale. Scores range from 13 to 65, with higher scores indicating higher parenting self-efficacy. In this study, Cronbach’s alpha was 0.80.

#### 2.3.3. Relationship with Smart Devices

The relationship between mothers and smart devices was evaluated in terms of maternal agency and affinity for these devices. Specifically, the study focused on two aspects: the degree of dependence on smart devices and confidence in selecting relevant parenting information. Participants were prompted with two questions: “Do you feel that you could not raise your child without a smart device?” and “Are you confident in your ability to discern and select useful parenting information obtained from your smart device?” Responses were recorded using a four-point Likert scale, ranging from 1 = “Disagree” to 4 = “Agree”.

#### 2.3.4. The Use of Smart Devices to Eliminate Parenting Concerns

Given the diverse contexts in which they used smart devices, participants were first asked to recall the most pressing concerns that they tried to resolve using their smart devices within the last three months. Response options included two items related to “Child’s health and development”, six related to “Child-rearing practices”, and “Other”. Participants were free to describe their concerns in detail. Second, we asked participants to rate the severity of the concerns they recalled on a scale of 1 = “trivial” to 6 = “serious”. Third, patterns in participants’ smart-device use were examined. Regarding social support classification [32], participants were asked whether they had made use of informational, emotional, or appraisal support. Fourth, we asked whether they used face-to-face advice as well as smart devices to resolve their concerns. Fifth, we asked about the degree of relief or anxiety that occurred as a result of using a smart device to resolve concerns. Responses ranged from 1 = “not at all reassured/not at all anxious” to 6 = “very reassured/very anxious”, respectively.

### 2.4. Statistical Analysis

We analyzed the subgroup of the two concerns (“child’s health and development” and “child-rearing practices”) that the participants attempted to resolve using the selected smart device. We believed that the actions involved in attempting to resolve the two concerns would be different in nature, given the different origins of the two concerns, with one originating with the child and the other with the mother. For each subgroup, structural equation modeling (SEM) was used to test a path model explaining the degree of relief and anxiety from smart-device use in a multifactorial manner, including parental self-efficacy. The parameters of the model were estimated by the maximum likelihood estimation method, and the model was considered to exhibit acceptable fit if the following criteria were met: a non-significant result of the χ^2^ test [33], the comparative fit index (CFI) and the Tucker–Lewis index (TLI) being greater than 0.95 [34], the root mean square error of approximation (RMSEA) being smaller than 0.05 [35], and the standardized root mean square residual (SRMR) being smaller than 0.08 [34]. JMP Pro 15.0 was used for statistical analysis. The significance level was set at *p* < 0.05.

### 2.5. Ethical Considerations

We provided written explanations in the survey regarding the participants’ right to refuse to answer the questions, anonymity, how their personal information would be handled, and that participation in the survey was voluntary. In our written request for research cooperation, we explained to participants that returning the questionnaires would be considered consent to the study. If the questionnaire was completed, it was implied that participants provided their consent to participate. This study was conducted with the approval of the ethical review board of the Faculty of Health Sciences, Hokkaido University (Approval Number: 20-2) and performed consistent with the principles of the Declaration of Helsinki.

## 3. Results

### 3.1. Descriptive Statistics

In total, 293 (58.6%, *N* = 500) questionnaires were returned. Thirty-five participants who did not answer all survey items and one participant who never used smart devices to eliminate parenting concerns were excluded. Thus, 257 participants were included in the analysis (valid response rate: 51.7%).

Table 1 presents the descriptive statistics for all variables in this study. “Child-rearing practices” *(n =* 146, 56.8%) and “Child’s health and development” (*n* = 103, 40.1%) covered the most pressing problems that respondents attempted to resolve using smart devices within the last three months, followed by “Other” (*n* = 8, 3.1%). Details of the concerns selected by the study participants are presented in Supporting Information S1. For the whole sample, the means of feeling scores after smart-device use were 4.19 (SD = 0.92) and 2.77 (SD = 1.20) for relief and anxiety, respectively.

### 3.2. Analysis of Structural Equation Modeling

Prior to path analysis using SEM, a correlation analysis with all variables was conducted. The correlation matrix for the major variables is presented in Supporting Information S2 and S3. The highest correlation coefficient among all variables was −0.69, which is below the recommended threshold of 0.70 [36], suggesting that the variables did not exhibit severe multicollinearity problems.

### 3.3. Factors Explaining Relief and Anxiety in Resolving Concerns Related to Child’s Health and Development

**Figure 3:**
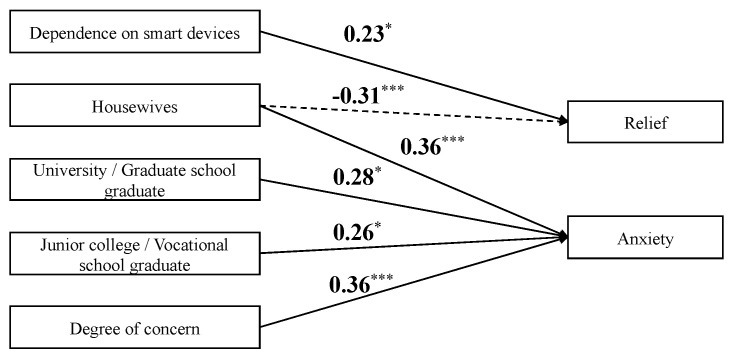
Results of the path model for child health and development. Note: Only exogenous variables and regression coefficients for which significant differences were found are depicted. Numbers are standardized coefficients. The solid arrows represent positive paths, and the dotted arrows indicate negative paths. All covariances have been omitted. * *p* < 0.05; *** *p* < 0.001.

The SEM results explaining relief and anxiety from the use of smart devices to alleviate concerns about children’s health and development are as follows: *χ*^2^(76) = 83.925, *p* = 0.250; CFI = 0.969; TLI = 0.952; RMSEA = 0.032; SRMR = 0.069. These indicators suggest that the constructed model represented good fit to the data. Table 2 presents the estimates of the paths from exogenous to endogenous variables in our model, Figure 3 depicts the significant paths, and Supporting Information S4 reports the estimates.

Significant explanatory variables for higher relief were not being a homemaker (*β* = −0.317, *p* = 0.001) and being more dependent on smart devices (*β* = 0.227, *p* = 0.013). Significant explanatory variables for high anxiety were being a homemaker (*β* = 0.369, *p* < 0.001), having a junior college or vocational school graduate degree (*β* = 0.261, *p* = 0.018), having a university or graduate school degree (*β* = 0.278, *p* = 0.010), and having serious concerns (*β* = 0.359, *p* < 0.001).

### 3.4. Factors Explaining Relief and Anxiety in Resolving Child-Rearing Practice Concerns

**Figure 4:**
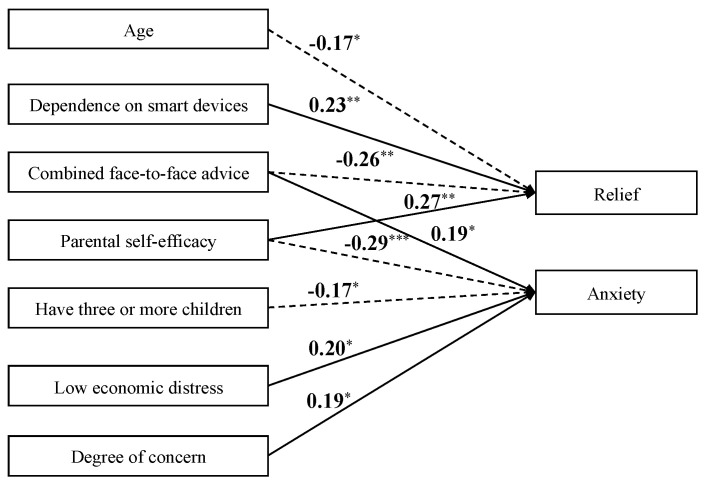
Results of the path model for child-rearing practices. Note: Only exogenous variables and regression coefficients for which significant differences were found are depicted. Numbers are standardized coefficients. The solid arrows represent positive paths, and the dotted arrows indicate negative paths. All covariances have been omitted. * *p* < 0.05; ** *p* < 0.01; *** *p* < 0.001.

The SEM results explaining relief and anxiety from smart-device use for reducing concerns about children’s health and development are as follows: *χ*^2^(69) = 70.425, *p* = 0.430; CFI = 0.996; TLI = 0.993; RMSEA = 0.012; SRMR = 0.062. These indicators suggest that the constructed model represented good fit to the data. Table 3 presents the estimates of the paths from exogenous to endogenous variables in the model, Figure 4 depicts the significant paths, and Supporting Information S5 presents all estimates.

Significant explanatory variables for higher relief were younger age (*β* = −0.169, *p* = 0.030), higher parenting self-efficacy (*β* = 0.272, *p* = 0.002), greater reliance on smart devices (*β* = 0.231, *p* = 0.005), and no combined face-to-face advice (*β* = −0.256, *p* = 0.002). Significant explanatory variables for high anxiety were not having three or more children, (*β* = −0.166, *p* = 0.037), not feeling economic deprivation (*β* = 0.197, *p* = 0.013), low parenting self-efficacy (*β* = −0.285, *p* = 0.001), serious concerns (*β* = 0.186, *p* = 0.017), and combined face-to-face advice (*β* = 0.188, *p* = 0.014).

## 4. Discussion

Comparing the relief caused by the use of smart devices to eliminate parenting concerns with the anxiety that also resulted indicated that the degree of relief was higher. Overall, mothers may experience positive psychological outcomes through the use of smart devices to solve parenting concerns. Since online parenting information is valued for its immediacy, practicality, and expertise [12], it likely helps to alleviate mothers’ concerns to some extent. However, this study also captured the reality of mothers who experienced anxiety owing to the use of smart devices. In addition, the seriousness of their concerns was found to be associated with mothers’ sense of anxiety.

This study showed that mothers with high parental self-efficacy tended to gain relief and feel less anxious when they used smart devices to resolve concerns regarding their parenting practices. Self-efficacy may help mothers use smart devices more effectively to solve parenting problems. Parental self-efficacy has been associated with lower postpartum anxiety levels [37] and reduced psychological distress [38]. Exposure to anxiety-provoking information [39] and psychological distress due to social comparisons [16] may be reduced by parental self-efficacy, resulting in mothers experiencing positive emotions with smart device utilization. Although the clinical significance of parenting self-efficacy has been identified [26], this study extended it to the context of smart-device use in parenting.

Contrary to concerns regarding parenting practices, there was a lack of association between mothers’ parenting self-efficacy and degree of relief and anxiety when they used smart devices to resolve concerns regarding child’s health and development. This discrepancy could be attributed to the contrasting nature of concerns centered around children and those centered around parenting practices. Parenting-practice-related concerns primarily focus on issues associated with skills and life knowledge, which can be progressively acquired over time. Consequently, these concerns are more likely to be linked with a sense of control for mothers who exhibit high self-efficacy in child-rearing. On the contrary, concerns regarding children’s health and development are more challenging for mothers to feel in control of, given the specialized knowledge often required to tackle such issues. Furthermore, compared to the worries about parenting practices, concerns over children’s health and development are more likely to be perceived as a higher threat. This is because these concerns are easier to envision in the context of the child’s life and well-being, thereby potentially inducing higher anxiety levels. Smart devices may serve as effective tools in addressing issues where the mothers perceive the threat level to be acceptable and controllable. This potential benefit of smart devices may be inferred from the results of the current study.

Participants who were homemakers gained less relief and experienced more anxiety when using their smart devices to resolve concerns about their children’s health and development. Homemaker mothers were more stressed and anxious [40] and more likely to experience exhaustion [41] than employed mothers. During the COVID-19 pandemic, homemakers were concerned about their psychosocial health and economic situation as well as parental fatigue [42]. Against this background, full-time homemakers’ concerns about their children’s health and development may grow. These concerns may not be resolved by means of smart devices.

Mothers with higher education levels were more likely to feel anxious because of the solutions to their concerns about their children’s health and development provided via smart devices. This result is surprising because highly educated parents tend to be more critical internet users [43], prefer authoritative sources such as professionals [44], and have higher e-health literacy [45]. This result may be because some highly educated mothers may have tried to use smart devices to address very difficult concerns, which in turn may have resulted in increased anxiety.

Mothers’ reliance on smart devices was associated with a higher degree of relief due to the elimination of parenting concerns through smart devices, but not with anxiety relief. The results suggest that smart devices are tools that positively influence parenting for mothers with a high affinity for smart devices. Parental problematic internet use (PIU) has been reported to be associated with child maltreatment [46] and child PIU [47], drawing attention to the problematic nature of parents’ reliance on smart devices in parenting. However, if not at the level of addiction, the use of smart devices by mothers who are rather dependent on smart devices may not be as problematic.

### 4.1. Implications

The fact that many participants experienced more positive than negative effects in using smart devices to resolve childcare concerns suggests that smart devices are useful childcare tools. Simultaneously, we must be mindful of the harmful effects of smart devices on parent–child interactions [48] as well as problematic parental internet use. However, parenting professionals should not be overly critical of smart-device use in child-rearing and should support parents in effectively using such devices. In addition, they may find it useful to focus on mothers’ parenting self-efficacy.

This study identified risk factors that may contribute to anxiety, induced by the use of smart devices to address childcare concerns. In this context, smart devices may not be suitable for homemakers, highly educated mothers, or serious parenting problems. The results also suggest that smart devices are less suited to resolving concerns about children’s health and development than child-rearing practices. Parenting professionals must disseminate information regarding such negative aspects of smart-device use to parents through their practices and policies.

### 4.2. Limitations

This study has some limitations. First, it adopted a cross-sectional design, and causal relationships could not be established. Second, recall bias was likely present. Although there is no guarantee that it was completely prevented, we attempted to minimize recall bias by encouraging free writing about the most pressing concerns addressed using smart devices in the preceding three months. Third, we may not have controlled for all predicted confounders. Smart-device use in child-rearing is expected to be strongly influenced by the mother’s media literacy and dependence on the device. There is no guarantee that these variables were accurately conceptualized and measured. Fourth, it is critical to acknowledge the limitations on generalization due to the specific context of this study. As this study was conducted during the COVID-19 pandemic, mothers may have had strong parental anxiety during this period [49], making it difficult to completely generalize the study’s findings in a post-COVID-19 world. Moreover, the valid response rate for our study approximated 50%, which, while commendable under the circumstances, introduced another potential source of bias.

Evidence on the effectiveness of smart-device use in child-rearing could be strengthened by extending the scope to parents with children of different ages than those in this study.

### 4.3. Conclusions

Smart devices are likely to be useful as problem-solving tools for parenting, and their usefulness is influenced by parental self-efficacy. Parental self-efficacy may have clinical significance in the context of smart-device use in parenting. Therefore, to encourage the helpful use of smart devices, parenting professionals should effectively strengthen support to increase parental self-efficacy.

## Figures and Tables

**Table 1:** Characteristics of mothers.

	Concern about Child’s Health and Development		Concern About Child-Rearing Practices		Full Sample	
	(*n* = 103)		(*n* = 146)		(*N* = 257)	
	**M (SD) or *n***	**%**	**M (SD) or *n***	**%**	**M (SD) or *n***	**%**
**Demographics**						
Age (years)	33.42 (4.58)	-	32.10 (4.39)	-	32.61 (4.47)	-
Number of children						
One	42	40.8	100	68.5	147	57.2
Two	42	40.8	35	24.0	79	30.7
Three or more	19	18.4	11	7.5	31	12.1
Occupational status						
Full-time workers	13	12.6	16	11.0	30	11.7
Part-time workers	12	11.7	6	4.1	18	7.0
Workers on maternity or childcare leave	35	34.0	62	42.5	101	39.3
Homemakers	43	41.7	62	42.5	108	42.0
Educational status						
Junior high school/high school graduate	21	20.4	27	18.5	50	19.5
Junior college/vocational school graduate	39	37.9	66	45.2	108	42.0
University/graduate school graduate	43	41.7	53	36.3	99	38.5
Subjective economic status						
Very concerned	4	3.9	5	3.4	12	4.7
Somewhat concerned	41	39.8	50	34.2	93	36.2
Slightly concerned	47	45.6	74	50.7	124	48.2
Not concerned at all	11	10.7	17	11.6	28	10.9
**Parental self-efficacy**						
Parenting self-efficacy scale (13–65)	48.31 (7.03)	-	48.09 (6.86)	-	48.12 (7.08)	-
**Relationship with smart devices**						
Dependence on digital devices (1–4)	3.17 (0.91)	-	3.36 (0.83)	-	3.28 (0.87)	-
Confidence in information selection (1–4)	3.03 (0.55)	-	3.02 (0.53)	-	3.02 (0.56)	-
**Use of smart devices to eliminate parenting concerns**						
Degree of concern (1–6)	4.24 (0.15)	-	3.77 (0.12)	-	3.98 (1.51)	-
How smart devices were used to eliminate concerns						
Informational support						
Yes	101	98.1	145	99.3	254	98.8
No	2	1.9	1	0.7	3	1.2
Emotional support						
Yes	46	44.7	56	38.4	109	42.4
No	57	55.3	90	61.6	148	57.6
Appraisal support						
Yes	38	36.9	55	37.7	97	37.7
No	65	63.1	91	62.3	160	62.3
Combined face-to-face advice						
Yes	92	89.3	94	64.4	190	73.9
No	11	10.7	52	35.6	67	26.1
Results from the use of smart devices						
Feeling relief (1–6)	3.97 (0.88)	-	4.40 (0.86)	-	4.19 (0.92)	-
Feeling anxiety (1–6)	3.05 (1.21)	-	2.55 (1.11)	-	2.77 (1.20)	-

**Table 2:** Factors associated with the degree of relief and anxiety caused by the use of smart devices to eliminate concerns about child health and development.

Exogenous Variables	Endogenous Variable											
	Relief						Anxiety					
	b	SE	95% CI		β	*p*	b	SE	95% CI		β	*p*
			LL	UL					LL	UL		
Parents’ demographics												
Age (years)	0.021	0.019	−0.017	0.059	0.104	0.283	−0.013	0.023	−0.058	0.033	−0.046	0.585
Number of children												
One	Reference						Reference					
Two	−0.147	0.191	−0.522	0.227	−0.082	0.441	0.025	0.229	−0.423	0.474	0.010	0.911
Three or more	0.087	0.246	−0.395	0.569	0.038	0.724	−0.562	0.294	−1.139	0.015	−0.175	0.056
Occupational status ^a^												
Workers	Reference						Reference					
Homemakers	−0.579	0.177	−0.926	−0.233	−0.317	0.001	0.941	0.212	0.526	1.356	0.369	<0.001
Educational status (highest educational qualifications)												
Junior high school/high school graduate	Reference						Reference					
Junior college/vocational school graduate	−0.408	0.239	−0.876	0.059	−0.219	0.087	0.679	0.286	0.119	1.239	0.261	0.018
University/graduate school graduate	−0.341	0.228	−0.788	0.106	−0.187	0.135	0.709	0.273	0.173	1.244	0.278	0.010
Subjective economic status ^b^	0.069	0.117	−0.160	0.298	0.055	0.556	0.175	0.140	−0.100	0.449	0.100	0.212
Parental self-efficacy												
Parenting self-efficacy scale	−0.006	0.012	−0.030	0.018	−0.045	0.632	0.022	0.015	−0.006	0.051	0.122	0.130
Relationship with smart devices												
Dependence on smart devices	0.225	0.091	0.048	0.403	0.227	0.013	−0.195	0.109	−0.408	0.018	−0.141	0.073
Confidence in information selection in smart devices	−0.009	0.156	−0.315	0.298	−0.005	0.955	−0.273	0.187	−0.640	0.094	−0.119	0.145
Situational factors												
Degree of concern	−0.091	0.055	−0.199	0.018	−0.147	0.101	0.311	0.066	0.181	0.441	0.359	<0.001
Utilizing for emotional support												
No	Reference						Reference					
Yes	−0.002	0.183	−0.361	0.357	–0.001	0.991	0.411	0.219	−0.020	0.841	0.163	0.061
Utilizing for appraisal support												
No	Reference						Reference					
Yes	0.270	0.193	−0.108	0.648	0.144	.161	0.067	0.231	−0.386	0.519	0.025	0.772
Combined face-to-face advice												
No	Reference						Reference					
Yes	−0.019	0.271	−0.550	0.512	−0.006	.943	0.501	0.324	−0.135	1.137	0.121	0.123
*R* ^2^	0.287						0.475					

Note: The estimates of paths from exogenous to endogenous variables in structural equation modeling are shown. *N* = 103; b = estimate; *SE* = standard error; *CI* = confidence interval; *LL* = lower limit; *UL* = upper limit; and β = standardized estimate. ^a^ “Full-time workers”, “part-time workers”, and “workers on maternity or childcare leave” were merged into “workers”. ^b^ Allocated 4 points for “Not concerned at all”, 3 points for “Slightly concerned”, 2 points for “Somewhat concerned”, and 1 point for “Very concerned”.

**Table 3:** Factors associated with the degree of relief and anxiety caused by the use of smart devices to eliminate concerns about child-rearing practices.

Exogenous Variables	Endogenous Variable											
	Relief						Anxiety					
	b	SE	95% CI		β	*p*	b	SE	95% CI		β	*p*
			LL	UL					LL	UL		
Parents’ demographics												
Age (years)	−0.033	0.015	−0.063	−0.003	−0.169	0.030	−0.004	0.019	−0.041	0.033	−0.016	.827
Number of children												
One	Reference						Reference					
Two	−0.145	0.160	−0.459	0.168	−0.072	0.364	0.092	0.196	−0.292	0.476	0.035	0.640
Three or more	−0.069	0.275	−0.608	0.470	−0.021	0.801	−0.701	0.336	−1.360	−0.042	−0.166	0.037
Occupational status ^a^												
Workers	Reference						Reference					
Homemakers	0.043	0.143	−0.238	0.324	0.024	0.766	0.012	0.175	−0.332	0.355	0.005	0.948
Educational status (highest educational qualifications)												
Junior high school/high school graduate	Reference						Reference					
Junior college/vocational school graduate	−0.011	0.182	−0.368	0.345	−0.007	0.951	−0.143	0.222	−0.579	0.293	−0.064	0.519
University/graduate school graduate	−0.037	0.210	−0.449	0.375	−0.020	0.860	−0.416	0.257	−0.920	0.087	−0.175	0.105
Subjective economic status ^b^	0.041	0.102	−0.159	0.242	0.034	0.687	0.310	0.125	0.064	0.555	0.197	0.013
Parental self-efficacy												
Parenting self-efficacy scale	0.035	0.011	0.013	0.057	0.272	0.002	−0.047	0.014	−0.074	−0.020	−0.285	0.001
Relationship with smart devices												
Dependence on smart devices	0.243	0.086	0.075	0.411	0.231	0.005	−0.131	0.105	−0.336	0.075	−0.096	0.213
Confidence in information selection in smart devices	−0.050	0.142	−0.328	0.227	−0.031	0.722	−0.334	0.173	−0.674	0.005	−0.159	0.054
Situational factors												
Degree of concern	0.039	0.047	−0.053	0.132	0.069	0.402	0.137	0.057	0.024	0.249	0.186	0.017
Utilizing for emotional support												
No	Reference						Reference					
Yes	0.115	0.157	−0.193	0.422	0.064	0.465	0.016	0.192	−0.360	0.392	0.007	0.934
Utilizing for appraisal support												
No	Reference						Reference					
Yes	0.260	0.163	−0.060	0.579	0.144	0.112	0.228	0.199	−0.163	0.619	0.098	0.254
Combined face-to-face advice												
No	Reference						Reference					
Yes	−0.463	0.147	−0.751	−0.175	−0.256	0.002	0.439	0.179	0.088	0.791	0.188	0.014
*R* ^2^	0.216						0.295					

Note: The estimates of paths from exogenous to endogenous variables in structural equation modeling are shown. n = 146; *b* = estimate; *SE* = standard error; *CI* = confidence interval; *LL* = lower limit; *UL* = upper limit; and β = standardized estimate. ^a^ “Full-time workers”, “part-time workers”, and “workers on maternity or childcare leave” were merged into “workers”. ^b^ Allocated 4 points for “Not concerned at all”, 3 points for “Slightly concerned”, 2 points for “Somewhat concerned”, and 1 point for “Very concerned”.

## Data Availability

The datasets generated during and/or analyzed during the current study are available from the corresponding author on reasonable request.

## Supplementary Materials

The following supporting information can be downloaded at: https://www.mdpi.com/article/10.3390/children10091437/s1, Supporting Information S1: Contents of concerns that parents attempt to eliminate using smart devices; Supporting Information S2: Correlations of variables in the model for concern about child’s health and development; Supporting Information S3: Correlations of variables in the model for concern about child-rearing practices; Supporting Information S4: All parameter estimates of structural equation modelling for concern about child’s health and development; Supporting Information S5: All parameter estimates of structural equation modelling for concern about child-rearing practices.
